# Take Your Vacation

**Published:** 1875-04

**Authors:** 


### TAKE YOUR VACATION.

This has been an unusually long and severe winter. Business and professional men have been compelled to work more industriously than usual by reason of the lull in the extensive business transactions of the country. When business is "dull," we are all apt to adhere to our stores and offices the more tenaciously, with a hope of securing patronage that would not likely be entrusted to subordinates. So it has come that we are all overworked in mind and body—have been urging our physical and intellectual machines without halting for repairs. It is now time to slow up for a season, and repair the extensive damage done in so long and unceasing a run. If we do not, certain enfeeblement or perhaps destruction of our machinery will ensue. We are forewarned that such rest is needed by the headaches, backaches, indigestion, sleeplessness, lassitude, and other bad feelings of which we find ourselves complaining. What had best be done? Seek a change of scene. Go visit your friends in the country, or the west. Go into the forest in quest of fish and game.—Go to the sea-side—go anywhere to procure change of scene, air and diet, but above all, *rest from business cares.*

We are often told that the idea of the modern generation that rest is required in the summer season, from business, is ridiculous, in view of the fact that our fathers and mothers did not deem watering places and sea-side resorts necessary to their healths and happiness. But it must be remembered that we live twice as fast, work twice as hard and earn twice as much money as did our ancestors, therefore, require twice the relaxation. If our grandfathers could wake up and take a hand with us in the business transactions of this day, we could send them to bed or to a lunatic asylum at the end of the first quarter. *Take your rest.*

